# Simultaneous Determination of Glibenclamide and Silymarin Released from Chitosan Microparticles by HPLC-ESI-MS Technique: Method Development and Validation

**DOI:** 10.3390/pharmaceutics14102164

**Published:** 2022-10-11

**Authors:** Mihaela-Iustina Condurache, Anca-Roxana Petrovici, Natalia Simionescu, Bianca-Stefania Profire, Luminita-Georgeta Confederat, Alexandra Bujor, Anca Miron, Lenuta Profire

**Affiliations:** 1 Department of Biomedical Sciences, “Grigore T. Popa” University of Medicine and Pharmacy of Iasi, 700115 Iasi, Romania; 2 Centre of Advanced Research in Bionanoconjugates and Biopolymers, “Petru Poni” Institute of Macromolecular Chemistry, 41A Grigore GhicaVoda Alley, 700487 Iasi, Romania; 3 Department of Internal Medicine, “Grigore T. Popa” University of Medicine and Pharmacy of Iasi, 700115 Iasi, Romania; 4 “Sfântul Spiridon” County Emergency Clinical Hospital, 700111 Iasi, Romania; 5 Department of Pharmaceutical Technology, “Grigore T. Popa” University of Medicine and Pharmacy of Iasi, 700115 Iasi, Romania; 6 Department of Pharmacognosy, “Grigore T. Popa” University of Medicine and Pharmacy of Iasi, 700115 Iasi, Romania; 7 Department of Pharmaceutical Chemistry, “Grigore T. Popa” University of Medicine and Pharmacy of Iasi, 700115 Iasi, Romania

**Keywords:** chitosan microparticles, glibenclamide, silymarin, HPLC-ESI-MS method development and validation

## Abstract

The study aim was to develop and validate a high-performance liquid chromatography–electrospray ionization mass spectrometry (HPLC-ESI-MS) method to simultaneously determine glibenclamide (Gli) and silymarin (Sil) released from chitosan (CS) microparticles in aqueous solutions. The CS microparticles were synthesized using an ionic gelation method, and their morphology, swelling degree, encapsulation efficiency and active substance release were investigated. Gli and Sil were loaded in different concentrations, and their identification and quantification were performed using the HPLC-ESI-MS method, which was further validated. The drugs’ characteristic m/z was found in the higher intensity of retention time (Rt) (Gli, 8.909 min; Sil A, 5.41 min; and Sil B, 5.66 min). The method selectivity and precision are very good, and the blank solution proved no interference. The linearity of the answer function is very good for Sil A (R^2^ = 1), Sil B (R^2^ = 0.9998) and Gli (R^2^ = 0.9991). For Gli, we obtained a limit of detection (LOD) = 0.038 mg/mL and limit of quantification (LOQ) = 1.275 mg/mL; for Sil A, a LOD = 0.285 mg/mL and LOQ = 0.95 mg/mL; and for Sil B, a LOD = 0.045 mg/mL and LOQ = 0.15 mg/mL. A high-resolution HPLC-ESI-MS method was developed and validated, which allowed the simultaneous determination of Gli and Sil loaded in CS microparticles, in a concentration range of 0.025–1 mg/mL.

## 1. Introduction

Diabetes mellitus (DM) is the biggest challenge of the 21st century for the public health system worldwide. DM therapy represents up to 10% of many countries’ health budgets, and DM prevention can substantially reduce the associated costs of clinical therapeutic and psycho-social impact [1]. There are currently approximately 1.1 million children and adolescents under 20 diagnosed with diabetes [2].

Type 2 DM (DM2) accounts for more than 90% of all DM cases and is characterized by hyperglycemia presence, as a result of reduced insulin secretion. Chronic hyperglycemia is associated with ocular microvascular complications, renal damage, peripheral neuropathy and cardiovascular and cerebral macrovascular complications [3].

Recent studies have aimed to develop drugs capable of protecting pancreatic beta cells from oxidative stress and the inflammatory process by controlling hyperglycemic conditions [4].

Recently, various therapeutic strategies have been used in the treatment of DM2 [5]; they are designed to ensure adequate glycemic control and, at the same time, to improve the chronic complications induced by this condition [6,7,8]. Conventional therapies for DM2 include sulfonylureas that stimulate insulin secretion and biguanides (metformin) that increase insulin sensitivity. Other therapeutic agents, more recently developed, include alpha-glucosidase inhibitors, thiazolidinediones, meglitinides (analogs of metiglinide) and modulators of incretins [9]. Alpha-glucosidase inhibitors act in the gastrointestinal tract by reducing glucose uptake [10], while thiazolidinediones target several intracellular metabolic pathways, resulting in increased insulin action and improved tissue sensitivity to this endogenous hormone [11]. Meglitinides, like sulfonylureas, work by stimulating insulin secretion from pancreatic beta cells [12]. Incretin modulators include glucagon-like peptide 1 (GLP-1) analogs, which stimulate insulin secretion, and dipeptidyl peptidase IV (DPP-IV) inhibitors, which prolong the activity of GLP-1 and synthetic analogs [13]. Relatively new categories of antidiabetic agents are sodium glucose co-transporter type 2 (SGLT-2) inhibitors, which are responsible for renal glucose reabsorption. Inhibition of this co-transporter causes glucosuria and thus may reduce hyperglycemia in diabetes patients [14].

Glibenclamide (Gli) is a second-generation sulfonylurea commonly used in DM2 treatment [15,16]. Gli treatment is an effective therapeutic strategy that successfully lowers blood glucose levels [16] and is also prescribed in combination therapy with metformin. The doses administered are relatively low compared to first-generation sulfonylureas. Sulfonylureas work by increasing the concentration of insulin, as a result of stimulating pancreatic beta cells, through inhibition of sensitive ATP potassium channels by binding to a specific subunit known as sulfonylureas’ receptors [17]. The result is inhibition of the flow of potassium ions with membrane depolarization, activation of voltage-gated calcium channels and increased inflow of calcium ions [17]. Increasing the intracellular concentration of calcium ions triggers the release of insulin from pancreatic beta cells through the fusion of insulin vesicles with the cell membrane and their subsequent exocytosis [17]. Due to their mechanism of action, sulfonylureas are more effective in the early stages of DM2, when the function of pancreatic beta cells is not affected [17].

The combination of antidiabetic drugs with silymarin is increasingly used in current medical practice due to its many beneficial effects [18,19,20]. Silymarin is one of the most widely used natural antioxidants due to its ability to neutralize free radicals, with proven beneficial effects on DM patients [21]. It has been demonstrated that silymarin has the ability to stimulate protein synthesis in the liver, which leads to a considerable increase in the renewal rate of liver cells [22,23].

Chitosan (CS) is a polycationic linear polysaccharide, the second most abundant natural polysaccharide after cellulose [24]. CS is a non-toxic, biocompatible and biodegradable biopolymer that has low immunogenicity and can be derivatized to amino groups [25]. Studies have also shown that CS has significant biological effects, such as antidiabetic [26], antioxidant, antitumor, antimicrobial, anti-inflammatory and cholesterol-lowering properties [27]. Due to its biological and physicochemical characteristics, CS is extensively studied for pharmaceutical and biomedical applications, including drug delivery systems [28].

Starting from the premise of developing new therapeutic systems containing an antidiabetic active substance (Gli) and a liver protector (Sil), it was necessary to optimize and validate a method capable of allowing simultaneous quantitative determination of these two classes of active substances used, released from chitosan microparticles, employing an HPLC-ESI-MS technique using an external calibration method. By analyzing the existing studies regarding the chromatographic separations of Gli [29] and Sil [30], we succeeded in the first phase of developing and optimizing a chromatographic method capable of achieving the proposed objectives. For more accuracy, the validation of the developed method was performed, following parameters such as linearity, selectivity, limits of quantification (LOQ) and detection (LOD), precision of the method and system, and last but not least the accuracy of the method. This method also allows for both in vivo and in vitro applicability.

## 2. Materials and Methods

### 2.1. Materials

Chitosan (CS, medium molecular weight 190–310 kDa, degree of deacetylation 75–85%), pentasodium tripolyphosphate (TPP, ≥85%), glibenclamide (Gli), silymarin (Sil), formic acid (CH_2_O_2_) 98–100%, HPLC-grade methanol and acetonitrile (ACN), taurocholic acid sodium salt (≥95%), lecithin (≥60%), pepsin from gastric porcine mucosa (≥250 units/mg powder), sodium chloride (NaCl; ≥99%), hydrochloric acid (HCl; 37%), maleic acid (≥99%) and sodium hydroxide (≥97%) were purchased from Sigma Aldrich (St. Louis, MO, USA).

### 2.2. CS-Gli-Sil Microparticle Synthesis

In order to obtain CS-Gli-Sil microparticles, different active substance concentrations (15, 22.5, 30 mg) were dissolved in 0.5 mL each of DMSO (Gli) and methanol (Sil) and then mixed with 3 mL CS 1.2% water solution. The active substance proportions versus CS (*w*/*w*/*w*) were calculated as 0.5:0.5:1; 0.75:0.75:1 and 1:1:1, respectively. The mixture was kept at 250 rpm for 2 h, followed by 30 min ultrasonication. The resulting solution was added dropwise to a 20 mL TPP 2% solution under slight agitation (250 rpm), using a syringe (26 G, 0.45 × 16 mm). The mixture was stirred for 5 h and then kept at room temperature for 12 h to complete the crosslinking process. Finally, the obtained CS-Gli-Sil microparticles were washed 3 times with distilled water to remove TPP and allowed to dry on a flat surface at room temperature until further determinations [31]. Separately, a 1.2% CS solution was prepared in water and 2% TPP was used as a crosslinking agent, leading to the formation of stable microparticles with optimal morphological characteristics.

### 2.3. Morphological Characterization of CS-Gli-Sil Microparticles

The environmental scanning electron microscopy (ESEM) studies were performed on samples placed on an aluminum support. The samples were covered with a carbon layer to ensure opacity to the flow of electrons. The coated surfaces were examined using an ESEM instrument, operating at 20 kV, at different resolutions. The diameter of the microparticles was calculated automatically, using the processing program supplied with the device.

### 2.4. Encapsulation Efficiency (EE%) and Drug Loading Percentage (DL%)

The active substance EE% in the CS matrix represents the amount of active substance embedded in the microparticles and was determined considering the specific spectral characteristics of the incorporated active substance. The EE% was calculated according to the following equation [32]:

$$\mathrm{EE}\;\left(\%\right)=\frac{m_{2}}{m_{1}}\times 100$$

where m_1_—initial active substance mass (mg) added into solutions; m_2_—active substance mass that has been encapsulated in microparticles (mg), determined from TPP solution after microparticle separation by using calibration curve equations. For each compound, the equation of the characteristic curve was used in order to determine the m_2_.

The DL% represents the active substance amount found in the final mass of CS microparticles and was calculated according to the following equation [32]:

$$\mathrm{DL}\;\left(\%\right)=\frac{m_{2}}{m_{1}}\times 100$$

where m_1_—final mass of CS-Gli-Sil, after drying (mg); m_2_—the encapsulated active substance mass (mg).

### 2.5. The Drug Releasing Degree DR (%) from the Polymer Matrix

The active substances’ (Gli, Sil) DR (%) from the polymeric matrix was studied according to a previously published method [32] with slight modifications. It used two releasing media: simulated gastric fluid (0.08 mM sodium taurocholate, 0.02 mM lecithin, 0.1 mg/mL pepsin, 34.2 mM sodium chloride and 25.1 mM hydrochloric acid, pH = 1.6) and simulated intestinal fluid (3 mM sodium taurocholate, 0.2 mM lecithin, 19.12 mM maleic acid, 34.80 mM sodium hydroxide and 68.62 mM sodium chloride, pH = 6.5), respectively.

In order to determine the in vitro active substance releasing degree in both simulated gastric and intestinal fluid, the experiments were performed as follows: First, 1 mg CS-Gli-Sil was incubated with 2 mL of simulated gastric fluid at 37–37.5 °C and 100 rpm for 2 h. Every 30 min, 1 mL of samples was collected and analyzed with the HPLC-ESI-MS method described below in order to determine the amount of active substances released. After 2 h, the microparticles were removed from the simulated gastric fluid and 2 mL of simulated intestinal fluid was added, and the experiment was continued for another 8 h. Every 60 min, 1 mL of samples was collected and measured according to the above procedure. After each analysis, fresh fluid (1 mL) was added in order to maintain reaction volume. The released concentrations were calculated based on the calibration curve equations of all the active substances used, and the active substances’ DR% from the polymeric matrix was determined using the following equation [31]:

$$\mathrm{DR}\;\left(\%\right)=\frac{c_{1}}{c_{0}}\times 100$$

where c_0_—initial active substance concentration in CS-Gli-Sil (mg/mL); c_1_—active substance concentration released at a given time interval (mg/mL).

### 2.6. The CS and CS-Gli-Sil Microparticle Swelling Degree (SD%)

In order to determine the CS and CS-Gli-Sil microparticle SD%, two media were used, distilled water and simulated gastric fluid, the latter having a pH of 1.2, as previously suggested [32].

#### 2.6.1. The CS and CS-Gli-Sil Microparticle SD% in Distilled Water

A 5 mL distilled water volume was added over 1 mg CS-Gli-Sil microparticles. Every 10 min during the first 2 h and then at 30 min, 60 min, 120 min and 24 h, the CS-Gli-Sil microparticles were separated from the aqueous medium, lightly dried with filter paper and weighed to determine the wet mass (m_0_). After weighing, the CS-Gli-Sil microparticles were reintroduced into the same distilled water volume, and the procedure was repeated until a constant mass was reached. Then, the CS-Gli-Sil microparticles were separated, dried at room temperature and weighed to determine the dry mass (m_1_). The SD%, which represents the amount of distilled water retained in the polymer matrix, was calculated according to the following equation [32]:

$$\mathrm{SD}\;\left(\%\right)=\frac{m_{0}-m_{1}}{m_{1}}\times 100$$

where m_0_—CS-Gli-Sil microparticles’ wet mass, determined at certain time intervals (mg); m_1_—CS-Gli-Sil microparticles’ dry mass, determined at the end of the experiment (mg).

#### 2.6.2. The CS and CS-Gli-Sil Microparticle SD% in Simulated Gastric Fluid

The experiment was performed using the same experimental protocol as described above, the difference being the use of the simulated gastric fluid. The experiment took place over 2 h, considering the physiological passage time in the gastrointestinal tract, and the weighing operation was performed every 10 min during the 2 h.

### 2.7. HPLC-ESI-MS Method Development

In order to develop the Gli and Sil in vitro and in vivo pharmacological study, the HPLC-ESI-MS technique must be used.

The analysis of Gli and Sil was performed using an Agilent 1200 Series HPLC system with a diode array detector (DAD) coupled to an Agilent 6520 accurate-mass quadrupole time-of-flight (Q-TOF) mass spectrometer (Santa Clara, CA, USA) equipped with an electrospray ionization (ESI) source. The separation was carried out on a 150 × 4.6 mm, 5 µm BDS Hypersil C18 column (Thermo Fisher Scientific, Waltham, MA, USA). The mobile phase consisted of MilliQ water with 0.1% formic acid (A), and HPLC-grade acetonitrile (B) was applied in a gradient (Table 1). Samples with a volume of 10 µL were injected with a flow rate of 1 mL/min, and the separation process ware monitored at 230, 280, 298 and 300 nm.

After the chromatographic detector, 0.1 mL/min was split and directed to ESI/Q-TOF MS. Nitrogen was used as a nebulizer (25 psi) and drying gas (7 L/min flow rate). The full ion scan in negative mode ranged from 50 to 1000 (m/z) at −4000 V ionization voltage and 325 °C. The mass scale was calibrated using manufacturer protocols and standards, and data were collected and processed using MassHunter Workstation Software Data Acquisition for 6200/6500 Series, version B.01.03.

Retention time (Rt) and MS chromatograms of Gli and Sil released from CS microparticles were confirmed by active substances’ standard curves.

#### Preparation of Standard Solutions

The standard curves were made by diluting 5 mg/mL Gli stock solution in methanol (HPLC grade) and 5 mg/mL Sil in MilliQ water in the 0.025–1 mg/mL range of concentrations, filtering the resulting solutions (0.22 μm filter) and subjecting them to HPLC-ESI-MS analysis using the above conditions. The peaks of interest considered were those of Gli and those of silibilin A (Sil A) and silibilin B (Sil B), major components of Sil. The peak areas (Y) of Gli, Sil A and Sil B standards versus concentrations (X) were plotted.

### 2.8. HPLC Method Development and Optimization

For the development and optimization of the HPLC analytical method aimed to establish the chromatographic conditions for Gli and Sil quantitative separation and determination, several gradient conditions, noted M1-M8, were studied (Table 1).

### 2.9. HPLC Method Validation

The best method for separating Gli and Sil was validated using the already published parameters [33,34]. The method validation was performed using standard solutions containing both Gli and Sil in the same concentration.

#### 2.9.1. Selectivity

The method selectivity was calculated following a protocol already published [34] and by using 1 mg/mL Gli and Sil standard solution and the chromatograms of a sample solution obtained after preparations of the CS-Gli-Sil microparticles, more exactly of the supernatant that contains the remaining unincorporated Gli and Sil, and of a blank solution containing no active substances. The selectivity factor (α) was calculated using the following formula:

$$\alpha =\frac{{\mathrm{Rt}}_{2}-t_{0}}{{\mathrm{Rt}}_{1}-t_{0}}$$

where Rt_1_ is the Sil A and Sil B Rt in standard solution; Rt_2_ is the Gli Rt in the standard solution; and t_0_ is the Sil A, Sil B and Gli Rt in the sample.

The method is considered selective if Gli, Sil A and Sil B peaks are not interfering with each other and if they have the same morphology as standard samples prepared in the same conditions [34].

#### 2.9.2. Precision

The system’s precision was calculated by injecting the same standard concentration (0.025, 0.05, 0.1, 0.3, 0.5, 0.7, 0.9 and 1 mg/mL) 6 times. The samples were filtered through a 0.22 µm filter and injected using M_8_ method chromatographic parameters and 10 µL injection volume. From the Rt determined for each of the 6 peaks analyzed, we calculated the standard deviation (SD) and the relative standard deviation (RSD%) which shows that the system used is precise if it is in the range of ±2% [33].

#### 2.9.3. Accuracy

In order to calculate the method accuracy, a combined stock solution of 0.5 mg/mL of Gli and Sil was taken into consideration, and 80, 100 and 120% proportions of the concentration were prepared individually from standards in the mobile phase and injected into HPLC-ESI-MS system 6 times using 10 µL injection volume and 1 mL/min flow. The results were processed according to the already published protocol [33].

#### 2.9.4. Linearity

The M_8_ method linearity was calculated using the concentration series representative of the Gli and Sil standard solutions, i.e., 0.025, 0.05, 0.1, 0.3, 0.5, 0.7, 0.9 and 1 mg/mL. All the samples were injected 6 times each, and the peak areas were calculated from the obtained chromatograms. The results were processed according to the already published protocol [34]. The linear regression curve and the corresponding equations of the active substances were obtained with the correlation coefficient (R^2^). By using the linear regression equation, the concentrations were determined and represented as a function of the theoretical concentration, and therefore the linearity of the results was obtained. If the correlation coefficient value (R^2^) is over 0.98, the calibration curve is considered linear [34].

#### 2.9.5. Signal-to-Noise Ratio

The signal-to-noise (S/N) ratio parameter was calculated using the system analysis software for each peak corresponding to Gli or Sil by taking into consideration the most appropriate Rt corresponding to background and Rt for each peak analyzed. The S/N ratio was automatically calculated by following the software’s instructions, as previously reported [34].

#### 2.9.6. Limit of Detection (LOD) and Limit of Quantification (LOQ)

LOD and LOQ were calculated as previously described [34], using the following formulas:

$$\mathrm{LOD}=3\times \frac{S}{N}\times lowest\;concentration\;of\;the\;linearity\;sample$$

$$\mathrm{LOQ}=10\times \frac{S}{N}\times lowest\;concentration\;of\;the\;linearity\;sample$$

## 3. Results

DM is a condition with an alarmingly increasing incidence in the last years [1]. In the current therapeutic approach for DM2, some of the most used drugs are those in the class of sulfonylurea derivatives. Due to the side effects generated by this compound such as hypoglycemia, gastrointestinal disorders, allergic reactions, hematological disorders, lactic acidosis and weight gain [3], a different approach regarding drug delivery systems must be undertaken. Sil, a product of plant origin, is known mainly for its regenerating and protective action on liver cells, forming a true shield against toxins and chemicals that can damage the liver. Furthermore, Sil has strong anti-inflammatory effects [35] and high antioxidant properties and has been shown to be beneficial in patients diagnosed with DM due to its ability to regulate blood sugar [36].

Below we describe the CS microparticles’ synthesis method as a drug delivery system of Gli and Sil, as well as the release profile of these two active substances. Gli (as a DM2 treatment drug) and Sil (as a liver protector) were incorporated into the same system, and their simultaneous release and quantification by the HPLC-ESI-MS method were investigated. At the same time, the method was validated, thus increasing the accuracy of the determinations.

### 3.1. CS-Gli-Sil Microparticles’ Synthesis

**Figure 1:**
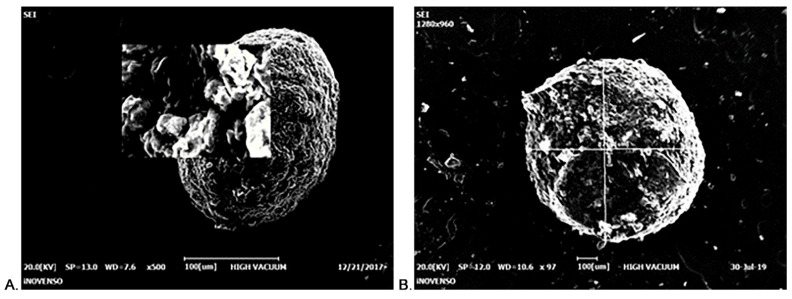
ESEM imaging: (**A**) CS microparticle; (**B**) CS-Gli-Sil microparticle.

CS-Gli-Sil microparticles were synthesized and characterized morphologically by ESEM (Figure 1). When the ratio between active substances (Gli and Sil) and CS was 1:1:1 (*w*/*w*/*w*), all the microparticles’ morphological and chemical characteristics were kept constant in solution and after separation from solution, in wet and dry states. CS microparticles are stable, having small sizes and a spherical, regular shape with a smooth surface (Figure 1A). Regarding the morphological aspects of CS-Gli-Sil microparticles analyzed by SEM, it was observed that they have an irregular shape with rough, slightly deformed surfaces (Figure 1B) compared to unloaded CS particles.

### 3.2. Encapsulation Efficiency (EE%) and Drug Loading Percentage (DL%)

**Figure 2:**
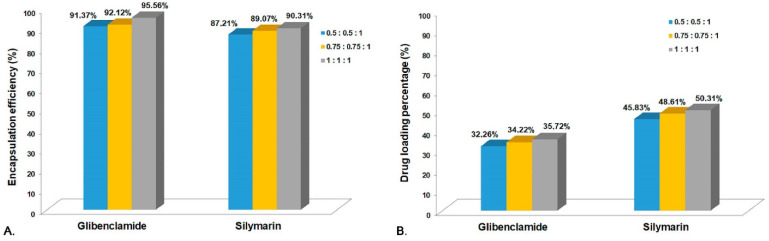
(**A**) Encapsulation efficiency for CS-Gli-Sil; (**B**) drug loading percentage for CS-Gli-Sil.

The active substances’ (Gli and Sil) EE% and DL% in the CS microparticles were determined based on the initial amounts used and the amounts remaining in TPP solutions after the system separation. In order to perform this experiment, three different proportions of active substances were used, and the quantification was performed using the developed HPLC-ESI-MS method (Figure 2).

In the analysis of the CS-Gli-Sil systems, a slight decrease in the encapsulation efficiency was observed in the case of Gli and Sil, the highest values being recorded at the highest weight proportion, 95.56% and 90.31%, respectively (Figure 2A). The DL% for both active substances was below 50% of the total system mass but increased with the active substances’ proportion, leading to almost 96% for Gli and 91% for Sil, respectively (Figure 2B).

### 3.3. The Drug Releasing Degree DR (%) from the Polymer Matrix

**Figure 3:**
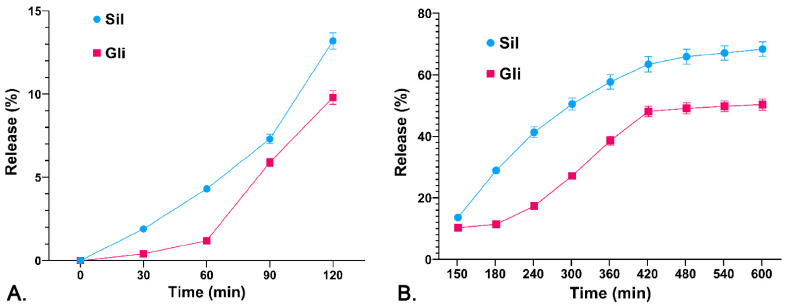
Release profile of the active substances (%) from the CS-Gli-Sil system: (**A**) in simulated gastric fluid; (**B**) in simulated intestinal fluid.

It was observed that the percentage of release from CS-Gli-Sil in the simulated gastric environment (the first 2 h) was relatively low, ranging between 10 and 12% (Figure 3A). The release intensifies substantially in the simulated intestinal environment, registering a steady growth over 8 h. Thus, the release percentage of Sil from CS-Gli-Sil was 68.30% and the release percentage of Gli was 50.34% (Figure 3B). Therefore, by incorporating Gli into the CS-Gli-Sil system, its bioavailability is increased, thus enhancing the efficacy of the treatment [37].

### 3.4. The Swelling Degree (SD%) of CS-Gli-Sil Microparticles

**Figure 4:**
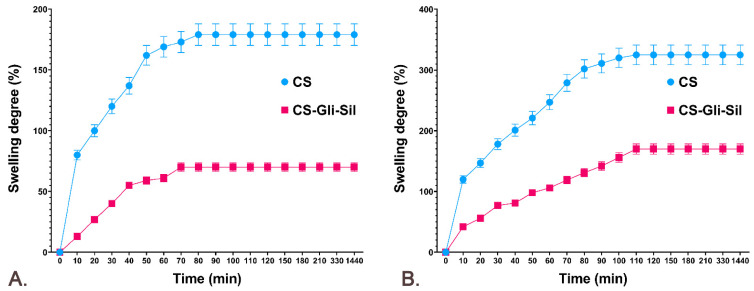
The CS and CS-Gli-Sil swelling degree (%): (**A**) in distilled water; (**B**) in simulated gastric fluid.

Since CS-Gli-Sil microparticles have been formulated for oral administration, the release of the active substances from the polymeric matrix and the bioavailability are influenced by both the degree of swelling and the porosity of the microparticles. In the analysis of the behavior of CS-Gli-Sil microparticles, it was observed that the largest amount of distilled water is absorbed in the first 10 min, after which the absorption proceeds more slowly. The balance is reached after about 70–90 min, depending on the active substance incorporated, and maintained for 24 h (Figure 4A). The swelling degree in distilled water was influenced by the physicochemical characteristics of the incorporated substances, having a value of 70% for CS-Gli-Sil after 80 min (Figure 4A).

The behavior of the microparticles in simulated gastric fluid was similar to that in distilled water. Therefore, the largest amount of simulated fluid was absorbed in the first 10 min, after which the absorption was slower. However, the balance was reached more slowly, after about 110–120 min, depending on the active substance incorporated, and was maintained for 24 h. For CS-Gli-Sil microparticles, higher values were recorded in simulated gastric fluid (170%) (Figure 4B) than in distilled water (70%) (Figure 4A).

### 3.5. HPLC-ESI-MS Method Development and Optimization

**Figure 5:**
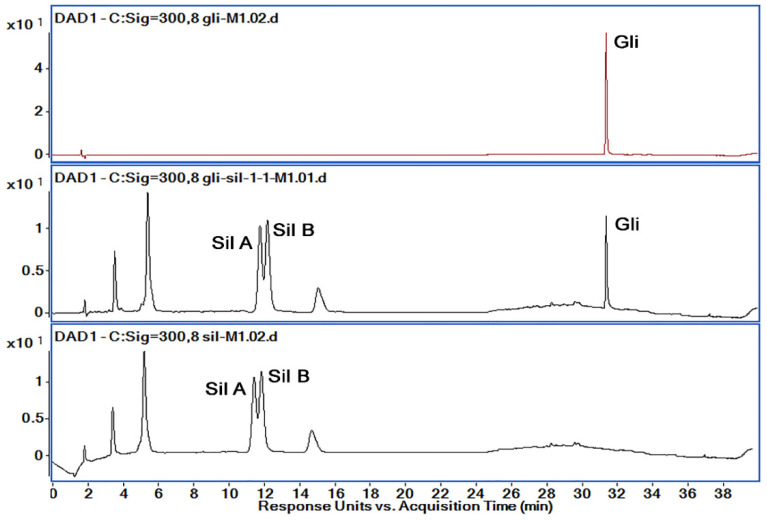
Chromatograms obtained by HPLC-DAD (300 nm) for the active substances’ mixtures using M_1_; retention times: Sil A 11.55 min, Sil B 12.32 min and Gli 31.9 min.

All methods used for the separation and identification of the active substances (Gli, Sil) are described in Table 1. Taking into consideration the retention times and organic phase proportion for Gli, Sil A and Sil B reported previously [29,33], we first chose a gradient method denoted M_1_ with a run of 40 min. Solutions of 1 mg/mL concentration of Gli, Sil and Gli + Sil were prepared, and all the samples were injected individually for peak Rt identification. From the spectra presented in Figure 5, the peak of interest was detected at 11.55 min for Sil A, at 12.32 min for Sil B and at 31.9 min for Gli.

**Figure 6:**
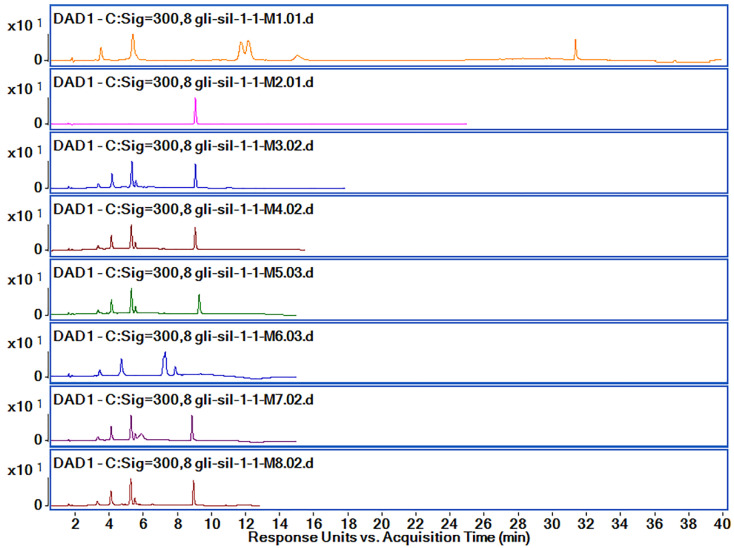
Chromatograms obtained by HPLC-DAD (300 nm) for the active substances’ mixtures using M_1_–M_8_ methods.

Analyzing the chromatograms obtained with M_1_ method, we observed that Gli was separated very well with a very good allure of the peak. However, the Gli peak Rt was too high for M_1_, and Sil A and Sil B peaks overlapped. Therefore, in order to obtain good peak separation and intensity, shorten the method’s run time and reduce solvent usage, we tried several variations of the method, presented in Table 1. Based on the obtained chromatograms corresponding to each method, presented in Figure 6, we chose the M_8_ method for further optimization and validation.

**Figure 7:**
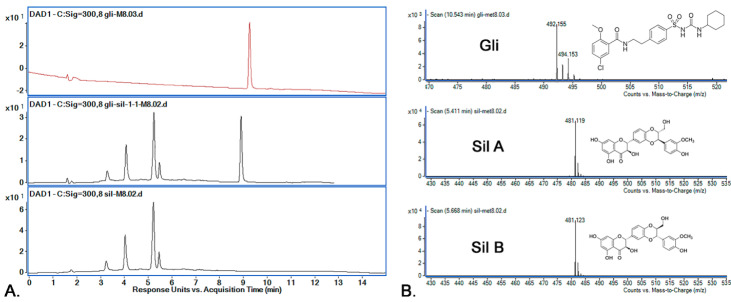
(**A**) Chromatograms obtained by HPLC-DAD (300 nm) for the active substances’ mixtures using M_8_; retention times: Sil A 5.41 min, Sil B 5.66 min and Gli 10.54 min; (**B**) active substances’ (Sil A, Sil B, Gli) identification using mass spectrometry.

The active substance identification was performed based on MS-MS spectra, as can be observed in Figure 7. The identification of the Sil A and Sil B peaks was performed based on previously published data [30], according to which the two peaks are separated one after the other. By using the M_8_ method for separation, the Rt for Sil A was registered at 5.41 min, for Sil B at 5.66 min and for Gli at 8.909 min. The m/z characteristic values for all active substances were found in the higher intensity for Rt presented above and are presented in Figure 7.

It was a big challenge to separate very well the two peaks of Sil A and Sil B that overlap for M_1_ and do not separate at all for M_2_. Korany et al. [30] report that the best peak separation of Sil A and Sil B is achieved when the organic phase is around 50%. However, we observed that the best separation occurred when the organic phase was fixed at 55% (M_3_–M_5_, M_7_, M_8_). When the mobile phase was set at 45%, the separation of Sil was not performed at all.

At the same time, we managed to shorten the Rt for Gli by modifying the gradient. According to Porwal et al. [29], Gli is best separated when the percentage of the organic phase is 55%, but we did not obtain a good resolution and therefore determined that it was necessary to increase the percentage (M_3_, M_4_, M_7_, M_8_). In order to reduce the cost of the analysis, the percentage of the mobile phase was increased to 60% (M_5_ and M_6_), but we observed that the intensity of Gli is lower, so the separation is worse. The best separation of all compounds was obtained by using M_8_, and the Rt and peak areas are presented in Table 2.

**Figure 8:**
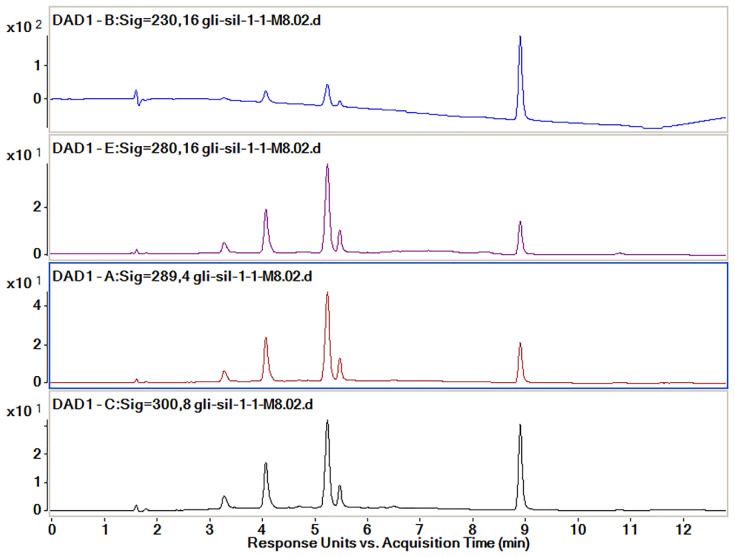
Chromatograms obtained by HPLC-DAD for the active substances’ mixtures at different wavelengths (230, 280, 298 and 300 nm).

In order to optimize the analysis method to efficiently separate the active substances, four simultaneous wavelengths (230, 280, 298 and 300 nm) at which all the spectra were recorded were analyzed (Figure 8). According to previously published data, Sil separates at 288 nm [30] and Gli separates at 227 nm [29]. When a chromatographic method is developed for the simultaneous separation of several different compounds, the spectra are also recorded at wavelengths close to those for which the maximum absorption is obtained because sometimes a good separation can be obtained at a wavelength close to that for which maximum absorption is recorded.

The wavelength used for the final calculation of the peak areas was established following the analysis of the data which are presented in Table 3. The proportion of each of the peaks of interest, namely those of Sil A, Sil B and Gli, was calculated from the sum of the areas of the three peaks recorded for each wavelength. The best proportion of peak areas and the best intensity of the peaks were determined to be those for the spectra recorded at 300 nm.

Analyzing all the data obtained from all analysis methods used, we concluded that the best separation method for all three compounds, namely Sil A, Sil B and Gli, is the M_8_ method with the extraction of spectra recorded at 300 nm.

### 3.6. Active Substances’ Calibration Curves

The standard curves were constructed using stock solutions of 5 mg/mL Gli in HPLC-grade methanol and 5 mg/mL Sil in MilliQ water. From these stock solutions, serial dilutions of Gli and Sil of 0.025, 0.05, 0.1, 0.3, 0.5, 0.7, 0.9 and 1 mg/mL were prepared and then filtered with a 0.22 µm filter and individually injected six times into the HPLC-MS system, using M_8_ parameters. The peaks of interest considered were those of Gli, Sil A and Sil B. The peak area was determined for all samples, and Table 4 shows the sum of all peak areas for all studied samples. Table 4 also presents the peaks’ asymmetry, resolution and selectivity, parameters which are calculated automatically by the HPLC-ESI-MS system software.

**Figure 9:**
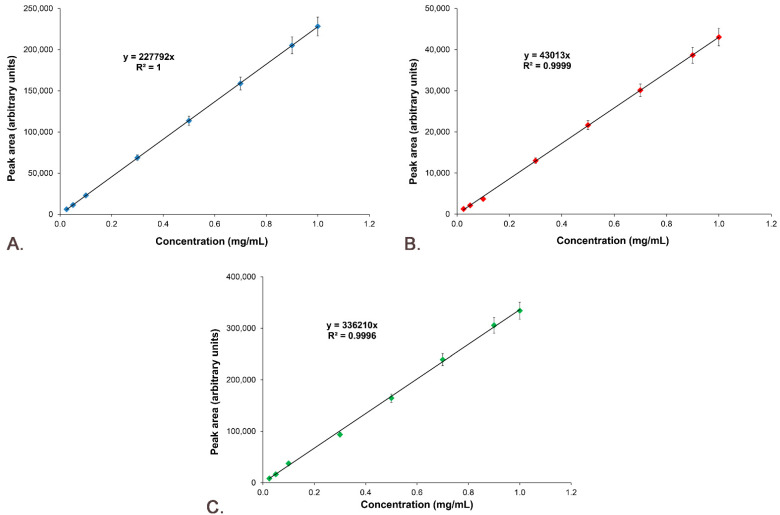
Standard calibration curves: (**A**) Sil A; (**B**) Sil B; (**C**) Gli.

The standard curves of Sil A, Sil B and Gli were represented by plotting peak areas versus concentrations, and the equations and R^2^ were represented for each curve (Figure 9).

### 3.7. M_8_ Method Validation

#### 3.7.1. Selectivity

**Figure 10:**
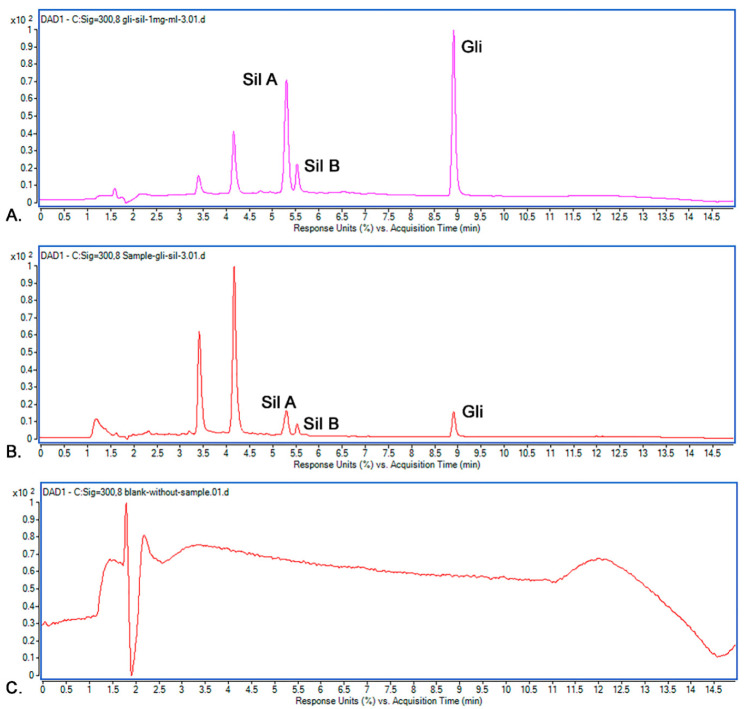
Chromatograms recorded: (**A**) standard solution; (**B**) sample solution; (**C**) blank solution.

The selectivity of the M_8_ method was studied by recording the Rt values for Sil A, Sil B and Gli from a standard solution and comparing them with Rt of the compounds from a sample solution against a blank solution. The injections were repeated three times, and the results’ accepted differences are within the maximum of 5%. Figure 10 shows the chromatograms for the standard solution, sample solution and blank solution.

It was observed that the recorded Rt values in the standard chromatogram for Sil A (5.28 min), Sil B (5.51 min) and Gli (8.909 min) were almost identical to the Rt values in the sample’s chromatogram: Sil A (5.28 min), Sil B (5.51 min) and Gli (8.906 min). Because the Rt values for all peaks from the standard and samples were the same and the blank solution proved no interference, we concluded that the selectivity of the methods is very good, and the M_8_ method allows the optimal separation of the studied active substances.

#### 3.7.2. Precision

The method’s precision was determined as was described before [34], and the SD% and RSD% for each concentration were calculated using the peak areas. Table 5 presents the Rt for the standard with a concentration of 0.5 mg/mL Gli and Sil. SD% and RSD% in the range of ±2% are characteristic of a precise method [33]. As can be observed in Table 5, the precision of the selected method is confirmed by the recorded values being lower than 0.09% for SD% and 0.9% for RDS%.

#### 3.7.3. Accuracy

The method’s accuracy was determined by considering 0.5 mg/mL Gli and Sil as 100% concentration. Solutions of 80% (0.4 mg/mL of Gli and Sil), 100% (0.5 mg/mL of Gli and Sil) and 120% (0.6 mg/mL of Gli and Sil) were prepared, as suggested by Rao et al. [33], following the protocol described in Section 2. The concentrations of Sil A, Sil B and Gli were calculated using the equations of the standard curves presented in Figure 9.

The calculated concentrations for Sil A, Sil B and Gli in proportions of 80%, 100% and 120% are presented in Table 6. The obtained data confirm the accuracy of the method used for compound quantification.

#### 3.7.4. Linearity

**Figure 11:**
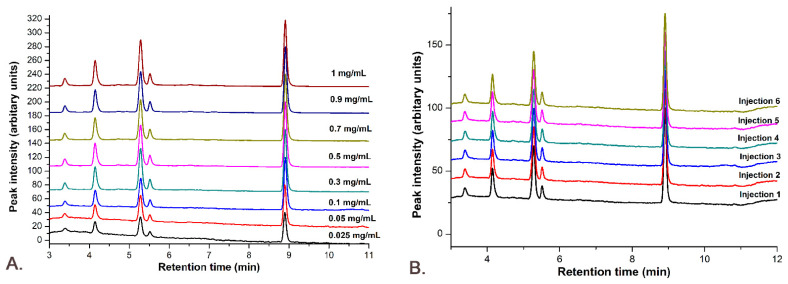
The M_8_ method linearity: (**A**) the chromatograms for all concentrations used in the calibration curve; (**B**) all 6 injections for the concentration of 0.1 mg/mL Gli-Sil.

The linearity was determined using the standard curve with the combined solution of Gli and Sil in concentrations of 0.025, 0.05, 0.1, 0.3, 0.5, 0.7, 0.9 and 1 mg/mL each. The standard solutions were injected six times each, and the peak areas were calculated from obtained chromatograms, presented in Figure 11.

The linearity was obtained through a graphical representation of the active compounds’ peak areas versus concentrations, plotting the calibration curve and determining the equation of the linear regression curve and the correlation coefficient (R^2^). Figure 9 demonstrates that the linearity of the answer function is very good for Sil A (Figure 9A) with an R^2^ value of 1, Sil B (Figure 9B) with an R^2^ value of 0.9998 and Gli (Figure 9C) with an R^2^ value of 0.9991.

**Figure 12:**
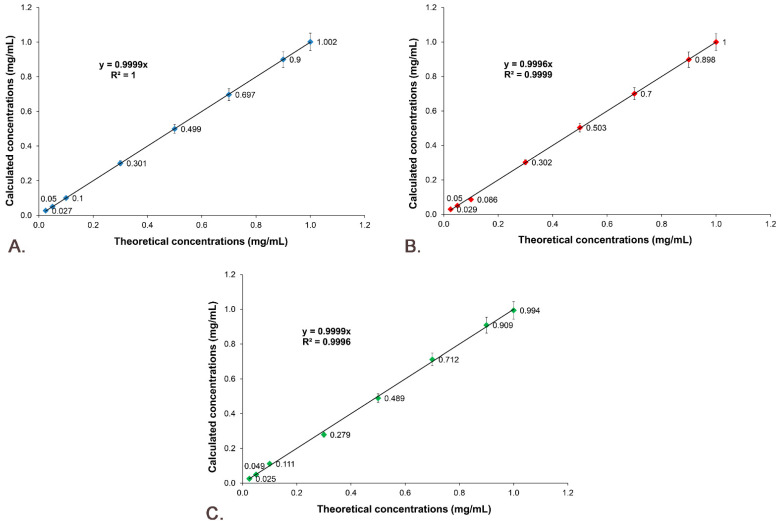
Linearity of the results: (**A**) Sil A; (**B**) Sil B; (**C**) Gli.

The linearity of the results for the studied active substances was obtained by representing the calculated concentration as a function of theoretical concentration, which is presented in Figure 12. The results prove that between the theoretical concentration and the calculated concentration there is a linear correlation, the R^2^ of the regression curves being 1 for Sil A (Figure 12A), 0.9998 for Sil B (Figure 12B) and 0.9991 for Gli (Figure 12C).

#### 3.7.5. Signal-to-Noise Ratio

The S/N was calculated using the protocol previously reported [34]. The set time interval for Sil A and Sil B was between 4.500 and 5.000 min before peaks and between 6.400 and 6.900 after peaks, and for Gli, the set time interval was between 8.100 and 8.600 min before the peak and between 9.300 and 9.900 min after the peak. The intervals were introduced in Agilent MassHunter Qualitative Analysis software and saved in the method editor, and all the S/N values were calculated automatically and are presented in Table 4. As can be observed, all the S/N values of the same compound, regardless of the analyzed concentration, are maintained around the same value.

#### 3.7.6. Limit of Detection and Limit of Quantification

The LOD and the LOQ for the studied active substances were calculated using the formula presented in Section 2.9.6. For Sil A, LOD = 0.285 mg/mL and LOQ = 0.95 mg/mL; for Sil B, LOD = 0.045 mg/mL and LOQ = 0.15 mg/mL; and for Gli, LOD = 0.038 mg/mL and LOQ = 1.275 mg/mL were obtained.

## 4. Conclusions

A system (CS-Gli-Sil) composed of chitosan microparticles loaded with an antidiabetic active substance, glibenclamide (Gli), and a liver cell protector, silymarin (Sil), was synthesized and characterized. The active substances’ encapsulation efficiency and the release profile make this drug delivery system suitable for further in vivo tests. In parallel, an HPLC-ESI-MS method capable of allowing the simultaneous separation and quantification of the active substances was developed and validated. The method is simple, selective and accurate and allows simultaneous quantitative determination of Gli and Sil from simulated gastric and intestinal fluids.

## Figures and Tables

**Table 1:** The chromatographic parameters used for HPLC method development and optimization.

Method	Gradient (% B)	Method Run Time
**M_1_**	0′–25; 12′–27; 22′–30; 26′–45; 31′–70; 37′–75; 40′–25	40
**M_2_**	0′–25; 5′–45; 10′–55; 15′–75; 20′–25; 25′–25	25
**M_3_**	0′–25; 5′–55; 10′–70; 20′–25	20
**M_4_**	0′–25; 5′–55; 10′–70; 12′–45; 15′–25	15
**M_5_**	0′–25; 5′–55; 10′–60; 12′–30; 15′–25	15
**M_6_**	0′–25; 5′–35; 8′–60; 10′–30; 15′–25	15
**M_7_**	0′–25; 5′–55; 8′–70; 10′–30; 15′–25	15
**M_8_**	0′–25; 5′–55; 9′–70; 12′–30; 15′–25	15

**Table 2:** The Rt and peak areas for studied compounds recorded for all methods (M_1_–M_8_).

Method	Sil Rt (Min)				Gli Rt (Min)		Observations
	Sil A		Sil B				
	Rt	Peak Area	Rt	Peak Area	Rt	Peak Area	
**M_1_**	11.69	105.4	12.12	117.7	31.36	61.8	Sil A and Sil B peaks are partially overlapping
**M_2_**	-		-		9.08	297.8	Sil is not separated at all
**M_3_**	5.29	180.2	5.52	30.9	9.0	151.5	poor separation of Sil B
**M_4_**	5.25	179.4	5.49	30.9	8.99	152.3	good peak separation
**M_5_**	5.25	178.2	5.49	31.0	9.23	150.3	separation of Sil A and Sil B peaks is not very good
**M_6_**	7.24	182.4	7.83	39	-	-	Gli peak is not separated
**M_7_**	5.23	176.5	5.47	28.4	8.81	149.8	individual peaks; efficient separation
**M_8_**	5.22	179.8	5.46	31.7	8.9	154.3	good peak separation and bigger peak area for all compounds

**Table 3:** The proportion of the peak area in relation to the total interest peak area.

Absorbance, nm	Sil Rt (Min)				Gli Rt (Min)		Observations
	Sil A		Sil B				
	Peak Proportion, %	Peak Area	Peak Proportion, %	Peak Area	Peak Proportion, %	Peak Area	
230	21.5	372.6	3.7	65.1	74.8	1312.1	Sil A and Sil B peaks are partially overlapping
280	66.1	223.5	12.2	41.1	21.7	73.5	Sil is not separated at all
289	63.1	271.8	11.6	50.1	25.3	108.9	Poor separation of Sil B
300	48.9	180.8	8.9	32.9	42.2	155.8	Equilibrated peak proportions

**Table 4:** The parameters recorded for all studied samples.

Sample	Rt (Min)	Peak Area	Asymmetry	Resolution	Selectivity	Start Rt (Min)	End Rt (Min)	S/N
**Gli-Sil** **0.025 mg/mL**	5.28	6079.4	0.866	1.833	1.068	5.11	5.43	3.8
	5.51	1254.4	1.248	25.237	1.935	5.43	5.70	0.6
	8.90	8266.8	1.023	7.339	1.139	8.77	9.11	5.1
**Gli-Sil** **0.05 mg/mL**	5.28	11,337.9	0.916	1.790	1.068	5.10	5.43	3.5
	5.52	2132.2	1.142	26.896	1.930	5.41	5.67	0.5
	8.90	16,414.7	1.125	4.433	1.073	8.73	9.11	5.1
**Gli-Sil** **0.1 mg/mL**	5.29	22,878.9	0.893	1.790	1.048	5.12	5.43	3.3
	5.51	3690.3	1.100	27.349	1.676	5.43	5.67	0.5
	8.90	37,369.6	1.169	6.532	1.092	8.76	9.13	5.3
**Gli-Sil** **0.3 mg/mL**	5.28	68,636.9	0.850	1.822	1.064	5.09	5.43	4.0
	5.51	12,994.7	1.153	26.690	1.880	5.43	5.70	0.7
	8.90	93,664.2	1.093	7.397	1.120	8.76	9.16	5.3
**Gli-Sil** **0.5 mg/mL**	5.28	113,633.6	0.858	1.834	1.072	5.06	5.43	3.7
	5.51	21,644.3	1.194	26.327	1.935	5.43	5.70	0.6
	8.90	164,515.4	1.081	5.918	1.096	8.76	9.12	5.3
**Gli-Sil** **0.7 mg/mL**	5.28	158,843.5	0.832	1.843	1.067	5.06	5.43	3.6
	5.51	30,121.0	1.192	26.627	1.939	5.43	5.69	0.6
	8.90	239,245.5	1.099	5.618	1.097	8.77	9.11	5.3
**Gli-Sil** **0.9 mg/mL**	5.28	205,027.6	1.247	1.754	1.068	5.07	5.43	4.0
	5.51	38,642.8	1.212	26.364	1.919	5.43	5.71	0.7
	8.91	305,778.9	1.086	7.745	1.115	8.77	9.13	5.6
**Gli-Sil** **1 mg/mL**	5.29	228,231.2	0.819	1.851	1.065	5.09	5.43	4.3
	5.51	43,027.7	1.222	26.347	1.917	5.43	5.70	0.7
	8.91	334,214.0	1.097	6.698	1.146	8.77	9.12	5.6

**Table 5:** The Rt calculated for all 6 injected samples, characteristic of a Gli and Sil concentration of 0.5 mg/mL.

Sample	Rt (Min)	Peak Area	Asymmetry	Resolution	Selectivity	Start Rt (Min)	End Rt (Min)	S/N	SD%	RSD%
**Gli-Sil** **(0.5 mg/mL) (1)**	5.28	113,386	0.979	1.749	1.069	5.05	5.43	3.8	0.017	0.154
	5.52	21,514.42	1.063	26.162	1.934	5.43	5.69	0.5	0.009	0.424
	8.91	163,446.6	0.947	4.732	1.07	8.77	9.1	5.4	0.075	0.459
**Gli-Sil** **(0.5 mg/mL) (2)**	5.2	113,515.5	0.975	1.881	1.095	5.06	5.43	3.7	0.008	0.073
	5.51	21,716.1	1.05	26.604	1.936	5.43	5.68	0.5	0.005	0.234
	8.9	163,444.7	1.006	2.516	1.075	8.753	9.106	5.2	0.075	0.460
**Gli-Sil** **(0.5 mg/mL) (3)**	5.29	113,633.8	0.788	1.856	1.067	5.08	5.43	3.7	0.001	0.001
	5.51	21,912.46	1.189	26.596	1.934	5.43	5.71	0.6	0.018	0.876
	8.91	166,850.12	1.121	7.803	1.117	8.73	9.18	5.3	0.165	1.003
**Gli-Sil** **(0.5 mg/mL) (4)**	5.28	113,677.68	0.91	1.844	1.067	5.05	5.43	3.7	0.003	0.027
	5.51	21,592.6	1.402	26.021	1.936	5.43	5.72	0.5	0.003	0.168
	8.9	164,387.06	1.205	7.079	1.111	8.77	9.12	5.2	0.009	0.055
**Gli-Sil** **(0.5 mg/mL) (5)**	5.28	113,729.7	0.798	1.862	1.067	5.05	5.43	3.6	0.006	0.059
	5.51	21,781.51	1.224	26.303	1.934	5.43	5.74	0.6	0.009	0.448
	8.9	164,049.2	1.102	7.46	1.107	8.77	9.11	5.3	0.032	0.200
**Gli-Sil** **(0.5 mg/mL) (6)**	5.28	113,858.7	0.814	1.857	1.067	5.05	5.43	3.7	0.015	0.140
	5.51	21,348.5	1.237	26.278	1.936	5.43	5.67	0.6	0.020	0.966
	8.9	164,914.8	1.107	5.916	1.098	8.771	9.11	5.2	0.028	0.171

**Table 6:** The calculated concentrations for accuracy determination of the method.

Samples	Samples Concentrations, mg/mL		
	Sil A	Sil B	Gli
**80%**	0.41	0.39	0.40
**100%**	0.49	0.49	0.50
**120%**	0.60	0.60	0.59

## Data Availability

The data presented in this study are available upon request from the corresponding author.
